# Natural Coumarins: Exploring the Pharmacological Complexity and Underlying Molecular Mechanisms

**DOI:** 10.1155/2021/6492346

**Published:** 2021-08-23

**Authors:** Javad Sharifi-Rad, Natália Cruz-Martins, Pía López-Jornet, Eduardo Pons-Fuster Lopez, Nidaa Harun, Balakyz Yeskaliyeva, Ahmet Beyatli, Oksana Sytar, Shabnum Shaheen, Farukh Sharopov, Yasaman Taheri, Anca Oana Docea, Daniela Calina, William C. Cho

**Affiliations:** ^1^Phytochemistry Research Center, Shahid Beheshti University of Medical Sciences, Tehran, Iran; ^2^Faculty of Medicine, University of Porto, Alameda Prof. Hernâni Monteiro, 4200-319 Porto, Portugal; ^3^Institute for Research and Innovation in Health (i3S), University of Porto, 4200-135 Porto, Portugal; ^4^Institute of Research and Advanced Training in Health Sciences and Technologies (CESPU), Rua Central de Gandra, 1317, 4585-116, Gandra, PRD, Portugal; ^5^Instituto Murciano de Investigación Biosanitaria (IMIB-Arrixaca-UMU), Clínica Odontológica Universitaria Hospital Morales Meseguer, Adv. Marques de los Velez s/n, 30008 Murcia, Spain; ^6^Lahore College for Women University, Lahore, Pakistan; ^7^Al-Farabi Kazakh National University, Faculty of Chemistry and Chemical Technology, Almaty 050040, Kazakhstan; ^8^University of Health Sciences, Department of Medicinal and Aromatic Plants, Istanbul 34668, Turkey; ^9^Department of Plant Biology Department, Taras Shevchenko National University of Kyiv, Institute of Biology, Volodymyrska Str., 64, Kyiv 01033, Ukraine; ^10^Department of Plant Physiology, Slovak University of Agriculture, Nitra, A. Hlinku 2, 94976 Nitra, Slovakia; ^11^Department of Plant Sciences, LCWU, Lahore 54000, Pakistan; ^12^Research Institution “Chinese-Tajik Innovation Center for Natural Products”, Academy of Sciences of the Republic of Tajikistan, Ayni 299/2, Dushanbe 734063, Tajikistan; ^13^Department of Toxicology, University of Medicine and Pharmacy of Craiova, 200349 Craiova, Romania; ^14^Department of Clinical Pharmacy, University of Medicine and Pharmacy of Craiova, 200349 Craiova, Romania; ^15^Department of Clinical Oncology, Queen Elizabeth Hospital, Kowloon, Hong Kong

## Abstract

Coumarins belong to the benzopyrone family commonly found in many medicinal plants. Natural coumarins demonstrated a wide spectrum of pharmacological activities, including anti-inflammatory, anticoagulant, anticancer, antibacterial, antimalarial, casein kinase-2 (CK2) inhibitory, antifungal, antiviral, Alzheimer's disease inhibition, neuroprotective, anticonvulsant, phytoalexins, ulcerogenic, and antihypertensive. There are very few studies on the bioavailability of coumarins; therefore, further investigations are necessitated to study the bioavailability of different coumarins which already showed good biological activities in previous studies. On the evidence of varied pharmacological properties, the present work presents an overall review of the derivation, availability, and biological capacities of coumarins with further consideration of the essential mode of their therapeutic actions. In conclusion, a wide variety of coumarins are available, and their pharmacological activities are of current interest thanks to their synthetic accessibility and riches in medicinal plants. Coumarins perform the valuable function as therapeutic agents in a range of medical fields.

## 1. Introduction

Plants synthesize a large collection of natural products called secondary metabolites [1, 2]. Secondary metabolites have important ecological functions, supporting plant protection against microbes and herbivores and participate in the attraction of pollinators etc. Humans use secondary metabolites as a source of drugs, aromatization, flavouring agents, and for a wide spectrum of other use [3]. Natural compound coumarins have been exposed to considerable phytochemical and pharmacological exploration in the last few decades. It is shown that over the last three years, over 400 coumarins have been described in scientific publications [4].

Coumarins are present naturally in a large number of plants, considerably in high concentration in *Coumarouna odorata* (tonka bean) (Fabaceae/Leguminosae) [5]. It is also present in high content in vanilla grass (*Anthoxanthum odoratum*) [6], sweet clover (genus *Melilotus*) [7], cassia cinnamon (*Cinnamomum cassia*) [8], in extracts of *Justicia pectoralis* [9], and a large number of cherry blossom trees [10]. Some species from the Apiaceae family (*Prangos* Lindl., *Ferula* L., *Heracleum* L., *Pachypleurum* Hoff., *Conioselinum* Fisch., *Libanotis* L., and *Seseli* L.) were rich with coumarin contents [11]. Many plants contain different concentrations of coumarin. Tonka beans, liquorice, and cassia cinnamon have a high concentration of natural coumarins. Some cherry blossom strawberries and apricots contain coumarin in smaller quantities. Despite its sweet smell, animals tend to avoid plants that contain coumarins due to their bitter taste [12]. Coumarins are natural bioactive compounds recognized for their anti-inflammatory, anticoagulant, antibacterial, antifungal, antiviral, anticancer, antihypertensive, antituberculous, anticonvulsant, antiadipogenic, and antihyperglycemic pharmacological activities, as well as its antioxidant and neuroprotective actions [13]. Over 1300 coumarins have been identified from plant sources. Tonka beans can contain, on average, 1% to 3% coumarin [14]. Animal studies have shown that these grains can be toxic, even in small amounts, causing serious liver damage in just a few weeks [15].

**Figure 1:**
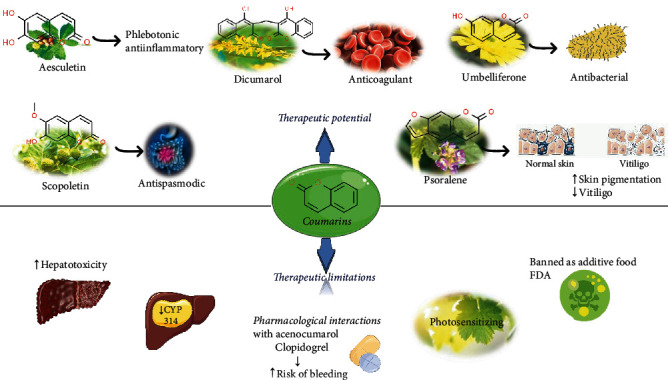
Diagram with the most relevant pharmacological properties related to chemical structures of natural coumarins and their derivatives.

Coumarins belong to the benzopyrone family commonly found in many medicinal plants (Figure 1). Their structure consists of two six-membered *rings* with lactone carbonyl groups. Most coumarin compounds are thermally stable and have notable optical activity [16]. The coumarin biosynthesis takes part in involving multiple P450 enzymes [17]. *Ortho*-hydroxylation is a key point for the biosynthesis of natural coumarins in plants [18].

**Figure 2:**
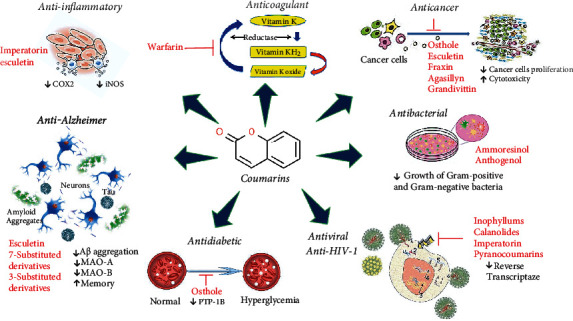
Schematic representation of molecular mechanisms and signaling pathways of the most representative coumarins and their derivatives. Abbreviations: COX-2: cyclooxygenase 2; iNOS: inducible nitric oxide synthase, PTP-1B: protein tyrosine phosphatase; MAO: monoamine oxidase.

Naturally occurring coumarins have been reported for their wide pharmacological activities, including antidiabetic [19], antioxidant, anabolic and hepatoprotective activities [19, 20], antifungal, and anti-inflammatory effects [21–23] (Figure 1). Calanolides, a natural coumarin, isolated from the *Calophyllum* genus have demonstrated strong anti-HIV activity [24] (Figure 2).

Accordance to evidence of varied pharmacological properties, the current paper presents an overall perspective of the derivation, availability, and biological capacities of coumarins with further consideration of the essential mode of their therapeutic actions.

## 2. Review Methodology

For this updated review, useful data were collected from the scientific databases PubMed, Science Direct, Scopus, Web of Science, and Elsevier, on the molecular mechanisms and pharmacological studies of natural coumarins and their derivatives. The following MeSH terms were used to search for them: “coumarins,” “chemistry,” “structure-activity relationship,” “pharmacology,” “drug therapy,” “humans,” “clinical trials,” “diabetes,” “anti –Inflammatory,” “neuroprotective,” “antibacterial,” “ antifungal,” and “antiviral.” The scientific names of the plants were verified using PlantList (http://www.theplantlist.org/), and the chemical formulas were verified with ChemSpider.

Inclusion criteria are as follows: preclinical pharmacological studies that highlighted the molecular mechanisms of action and signaling pathways and experimental *in vitro* and *in vivo* pharmacological studies.

Exclusion criteria are as follows: studies without obvious mechanisms and doses, works that were not written in English, and studies that included homeopathic preparations.

## 3. Bioavailability of Coumarins

Bioavailability is described as “the extent and rate to which the active drug ingredient or active moiety from the drug product is absorbed and becomes available at the site of drug action.” Usually, bioavailability mentions to the absorption of a compound from the gastrointestinal tract following oral administration of a dosage form [25, 26]. Therefore, it is not enough to know the dosage of taken compound(s); the more important issue is how much of that present is bioavailable. However, compound concentrations regularly cannot be determined directly at the site of action. Therefore, many bioavailability examinations concern to determine the concentration of the active compound in the blood or urine. Thus, bioavailability is involved with how fast and how much of a compound appears in the blood after a specific dose is administered.

A pharmacokinetic study was carried out by Ritschel and Hoffmann [27] who have compared between prolonged-release of coumarin containing tablets with intravenous and peroral administration of coumarin solution in humans, where a correlation was established between the percent of drugs released *in vitro* and the area under the curve (AUC). Xie and colleagues' [28] study showed that coumarin-based prodrug system produced a fourfold growth in oral bioavailability over the parent drug meptazinol in rats. In another kinetic study, Abraham et al. [29] presented that the absorption of coumarin from cinnamon powder is slightly lower than that of isolated coumarin.

In another research, the exchange of chloride anion by coumarin-3-carboxylate in sertraline, an antidepressant drug, enhances the pharmacological activity of the native drug, binding to bovine serum albumin with higher effect and saving the antimicrobial properties of the antidepressant molecule [30]. Newly designed coumarins can be oppressed for suppression of P-gp-mediated efflux to promote bioavailability of paclitaxel and can suppress breast cancer stem cell development which is crucial for planning potent anticancer drugs [31].

There are very few studies on the bioavailability of coumarins; therefore, future pharmacological studies are needed to study the bioavailability of naturally pharmacologically active coumarins.

## 4. Natural Coumarins and the Link between Chemical Structures and Pharmacological Effects

Coumarin molecules are made of combined benzene and *α*-pyrone rings and are chemically 2H-1-benzopyran-2-one which involves a large group of phenolic constituents in plants. The term coumarin derives from Coumarona odorata, a South American vegetable from which the molecule was first isolated in the 1820s. Coumarins were originally isolated in *Dipteryx odorata* Wild (tonka bean) [32].

They have been observed in over 150 various plants which belong to almost 30 diverse families, of which some important ones are Apiaceae, Caprifoliaceae, Clusiaceae, Guttiferae, Nyctaginaceae, Oleaceae, Rutaceae, and Umbelliferae. Naturally occurring coumarins are chiefly grouped into six types. In Figure 1 and Table 1, different types of coumarin are listed with their reported pharmacological activities.

At the end of the 19th century, farmers in North America introduced sweet clover (*Melilotus officinalis*) to animal feed and soon a hemorrhagic epidemic arose, which later proved to be correlated with the consumption of sweet clover. During the drying of the clover, coumarin undergoes a series of chemical transformations—partly spontaneous and partly mediated by Aspergillus fungi resulting in dicumarol [56]. Dicumarol interferes with the blood clotting process, blocking the synthesis of vitamin K-dependent clotting factors. One of its chemical derivatives, warfarin, is currently used as an oral anticoagulant in the treatment of deep vein thrombosis, prophylaxis of pulmonary embolism, myocardial infarction in patients with atrial fibrillation, or valve prostheses. Another chemical derivative of dicumarol, acenocoumarol, the active ingredient in sintrom drug, has similar therapeutic indications [57].

Scopoletin from the species Viburnum prunifolium and Angelica (essential oil extracted from the roots) has hypotensive and spasmolytic properties—able to inhibit the spastic contraction of smooth gastrointestinal and genitourinary muscle [58]. Khelline and visnagine, two coumarins of the species *Ammi visnaga*, have spasmolytic action on the smooth muscle of the coronary vessels with an antianginal effect [59]. Umbelliferone, a coumarin present in the grassy aerial parts of *Pilosella* and the resins of many Umbelliferae species, is used as a sunscreen and has antibiotic properties against brucellosis [60].

Esculetin, a coumarin present in chestnut leaves (*Aesculus hippocastanum*) due to the aglycone esculetin decreases the permeability of capillaries and increases their resistance, has anti-inflammatory and phlebotonic effects, improving venous tone. The same coumarin, from the flowers of the sweet clover (Melilotus officinalis), demonstrated antiedematous properties in vivo using animal models. Due to these properties, esculetin in combination with flavonoids is recommended as a phlebotonic adjuvant in the treatment and prophylaxis of chronic venous insufficiency [61]. Esculetin has also bacteriostatic and antifungal properties, while dafnoretin and 3-phenylcoumarins have antiviral properties against hepatitis B virus and anti-HIV. Esculetin also inhibits the synthesis of prostaglandins, thromboxanes and leukotrienes, and proinflammatory molecules involved in asthmatic, allergic, and inflammatory reactions [62].

Phytoalexins are plant-derived coumarins with general protective properties to fungal infection, physical injury, chemical damage, or a pathogenic progression. The main function of phytoalexins is to constrain or destroy the attacking agents like bacteria, viruses, and insects. Ayapin (6,7-methylenedioxycoumarin) is phytoalexin which was initially isolated from *Eupatorium ayapana*, a member of Asteraceae. Later on it was also isolated from several plants such as *Artemisia apiacea* [63], *Helianthus annuus*, *Pterocaulon polystachyum* [64], and *Pterocaulon virgatum* [65].

In plants, coumarin can be found in both free and glycosidic form, bound in the form of an aglycone to a glucose molecule. The high structural heterogeneity of coumarins justifies the great pharmacological variability with benefits for human health.

## 5. The Mechanisms behind the Pharmacological Activities of Natural Coumarins

### 5.1. Anti-Inflammatory Activity

Inflammation starts with five major signs: pain, swelling, redness, heat, and loss of function which ended up in vascular permeability, leukocytes invasion, production of pain, local oedema, and necrosis [66, 67]. Inflammation can be a denotation of multiple diseases like osteoarthritis, Alzheimer, and atherosclerosis [3]. Moreover, many types of cancers are related to chronic inflammation. Therefore, it is important to deal with inflammation before it turns up into a severe disease [68, 69].

Coumarin possessed anti-inflammatory characteristics, and its mechanism of action involves phagocytosis, production of the enzyme, and proteolysis to eradicate protein and oedema fluid from wounded tissue. One of its derivatives imperatorin also reported for its anti-inflammatory property in lipopolysaccharide-stimulated mouse macrophage (RAW264.7) *in vitro* as well as in carrageenan-induced mouse paw oedema model *in vivo*. It stops the protein expression of inducible cyclooxygenase-2 and NO synthase in lipopolysaccharide-stimulated RAW264.7 [70], whereas another derivative esculetin showed its anti-inflammatory potential in rat colitis induced by trinitrobenzene sulfonic acid [34, 71] This coumarin was extracted from *Bougainvillea spectabilis* and *Cichorium intybus*.

Coumarins possess antioxidant properties that may contribute to their anti-inflammatory effects [72]. The coumarin 1,2-benzopyrone has long been known to be effective in slowly reducing shoulder lymphedema. The underlying mode of action is unclear, but macrophage-induced proteolysis of the oedema proteins may be implicated.

### 5.2. Anticoagulant Activity

A complex system controls the blood fluidity in the human body [73]. Blood should remain inside the vasculature and also must clot quickly on exposure to injury [74]. Vitamin K is denoted as a blood anticoagulant, and the structure of warfarin is quite similar to this vitamin. Thus, warfarin is regarded as a vitamin K antagonist because of this structural and functional similarity [75].

Initially, warfarin was used as a poison for rat extermination during laboratory experiments; however, for the past 60 years, it was regarded as an anticoagulant. By mechanism, it inhibits the production of vitamin K-dependent coagulation factors (II, VII, IX, and X) in the liver microsomal. Vitamin K possibly induces the vitamin K-dependent coagulation factors (II, VII, IX, and X), N-terminal glutamate remains to form *γ*-carboxyglutamates. The reduced vitamin K (Vit KH2) contributes to the *γ*-carboxylation, and warfarin constrains the epoxide reductase to hinder the Vit KH2 development [75] (Figure 2).

### 5.3. Anticancer Activity

Cancers are characterized by an uncontrolled division of a group of cells, with the ability to invade other tissues in the body, either by a direct growth in those tissues or by the migration of cells to more distant places in the body (metastasis) [76]. In most cases of cancer, no specific cause of the disease can be identified [77]. Risk factors for cancer include exposure to anthropogens, chemicals, eating habits, and lifestyle [78].

In a recent study, coumarin was reported as a protective agent for mucosa and salivary glands in those patients who are undergoing head and neck radiotherapy [79]. Coumarin also can directly deal with cancer as osthole is active in controlling the migration and attack of breast cancer cells by healing the wound or transwell assays. Luciferase and zymography methods have shown that osthole efficiently hinders matrix metalloproteinase-s promoter and enzyme action, which could be one of the reasons that tend the relocation inhibition and attack by osthole [80], whereas esculetin is reported for its antitumor effects [81] and saves cultured primary neurons from N-methyl-D-aspartate toxicity [82].

Fraxin's defensive properties have been observed in the protection of cytotoxicity prompted by hydrogen peroxide in endothelial cells of the human umbilical vein [41]. Maximum of the coumarins grandivittin, agasyllin, aegelinol benzoate, and osthole displayed marginally cytotoxic effect across the A549 lung cancer cell, and they are usually extracted from *Ferulago campestris* [49].

### 5.4. Antibacterial Activity

It had been observed that pathogenic resistance against pharmaceutical and natural antimicrobial agents has been increased in recent times [83, 84]. Therefore, new prototype combinations are required to address this condition [85, 86]. In this regard, plant-based natural antimicrobial agents combined with antibiotics had shown a good potential [87, 88] Coumarins had been reported for combating against both Gram-positive and Gram-negative type of bacteria [89]. Long-chain derivatives of coumarin like ammoresinol and ostruthin showed more efficacy against *Bacillus megaterium*, *Micrococcus luteus*, *Micrococcus lysodeikticus*, and *Staphylococcus aureus.* However, another type of coumarin, i.e., anthogenol (extracted from *Aegle marmelos*) [90], displays a potent effect against *Enterococcus*. Moreover, a furanocoumarin compound imperatorin, which is extracted from *Angelica dahurica* and *A. archangelica* (Umbelliferae) [91], controls the growth and spread of *Shigella dysenteriae* [92]. A substantial antibacterial effect against clinically extracted Gram-positive and Gram-negative bacterial strains (such as *Staphylococcus aureus*, *Salmonella typhi*, *Enterobacter cloacae*, and *E. aerogenes*) was reported in *aegelinol* and *agasyllin*.

### 5.5. Antifungal Activity

An antifungal coumarin derivative, i.e., osthole, was extracted from *Angelica pubescens*, *Cnidium monnieri* [93], and *Peucedanum ostruthium* [94]. It showed a broad range of fungicidal property against microorganisms like *Botrytis cinerea*, *Fusarium graminearum*, *Phytophthora capsici*, *Rhizoctonia solani*, and *Sclerotinia sclerotiorum* [36]. After multiple antifungal experimentations, three most efficient have been reported, i.e., psoralen, imperatorin, and ostruthin [17].

### 5.6. Antiviral Activity

Coumarin is one of the herbal products which possess anti-HIV capabilities [95]. Inophyllums and calanolides (coumarin derivatives) presented new HIV inhibitory potential. Inophyllums A, B, C, E, P, G1, and G2 were extracted from *Achatina fulica*. Inophyllums B and P are reported to stop the activity of HIV reverse transcriptase with IC_50_ values of 38 and 130 nM, respectively. Moreover, both were also found effective against HIV-1 in cell culture (IC_50_ of 1.4 and 1.6 *μ*M) [51].

Calanolides A and B were extracted from the foliar parts of *Calophyllum lanigerum*. Studies reported both calanolides A and B as totally protective against replication of HIV-1 [52]. The (+)-calanolide A is a nonnucleoside reverse transcriptase suppressant with the potential efficacy against HIV-1. However, (−)-calanolide B and (−)-dihydrocalanolide B showed antiviral activities comparable to (+)-calanolide A [53, 96]. Formerly inophyllum A and (−)-calanolide B were derived from the seed oil of *Calophyllum inophyllum* Linn and *C. cerasiferum* Vesque, correspondingly. Both of them are members of the family Clusiaceae and known for effective HIV-1 reverse transcriptase blockers [97]. Pyranocoumarins (pseudocordatolide C and calanolide F) were extracted from *Calophyllum lanigerum* var. *austrocoriaceum* and *C. teysmannii* var. *inophylloide* (King) P. F. Stevens (Clusiaceae). Both of these compounds demonstrated anti-HIV effect properties [54], whereas imperatorin also reported inhibiting both vesicular stomatitis and gp160-enveloped recombinant HIV-1 infection in numerous T-cell and HeLa cell lines [98].

Coumarin-linked benzoxazole-5-carboxylic acids showed the inhibitory activity against hepatitis virus RNA polymerase. Inhibiting effect against hepatitis virus RNA polymerase can be demonstrated by coumarin-linked benzoxazole-5-carboxylic acids. Currently, it has been discovered that benzoxazole is conjugated with a coumarin with this methylene linker which displayed strong inhibitory activities on the hepatitis virus [99].

The coumarins have been found to exert antiviral activities including an interesting potential activity against the hepatitis C virus [100]. Likewise, different coumarins such as novobiocin and its analogues have shown antimicrobial activity. Novobiocin, extracted from *Streptomyces niveus*, is principally effective against Gram-positive bacteria. This drug is little used, however, due to the high incidence of associated adverse reactions and the frequent appearance of resistant strains.

### 5.7. Neuroprotective Effect

Neurodegenerative diseases are characterized by progressive dysfunction and neuronal loss, due in most cases to the accumulation of proteins with altered physicochemical properties [101]. This process causes the death of neurons in which it accumulates and the alteration of neural connections, influencing movement, speech, memory, intelligence, and other brain functions, following the areas where changes occur in the central nervous system [102].

Alzheimer disease is the degenerative disorder of the CNS (central nervous system) which is distinguished by weakening in memory, intellectual poor operation, personality alteration, and language obstruction [103, 104]. The prevalence of this disease is increasing day by day, and it is most common in old aged people [105]. Researches in recent times showed that coumarin and its derivatives can inhibit this disease [106].

A researcher group of Matos synthesized a sequence of 3-substituted coumarin derivatives, which work as an active inhibitor of monoamine oxidase A and B (MAO-A and MAO-B) isoforms and acetylcholinesterase (AChE) [107].

Esculetin showed neuroprotective action on cerebral ischemia damage in a middle cerebral artery-blocking model in mice at 20 *μ*g/mL [108].

*Coriandrum sativum* (coriander) is an essential medicinal plant containing coumarin as an effective component with neuroprotective effects that improve memory. A series of coumarin 7-substituted derivatives have been developed to demonstrate the inhibitory properties of choline esterase and monoamine oxidase B (MAO-B) [109]. In this regard, it has been reported that various coumarin derivatives selectively inhibit the enzyme MAO-B, implicated in Alzheimer's disease, and prevent A*β*1-42 aggregation, with low toxicity in the studied cells lines. These results suggest possible applications in the management of Alzheimer's disease.

### 5.8. Antidiabetic Properties

There is a clear association between diabetes mellitus and cardiovascular complications, and in this regard, the coumarins would be even more valuable if they were found to be effective against diabetes. In theory, these drugs would not only lower blood glucose but also improve the outcomes in terms of the cardiovascular complications of diabetic patients. In this regard, different studies have found plant extracts containing coumarins to exhibit antidiabetic activity [110]. Osthole containing volatile oil from roots of *Prangos pabularia* significantly inhibited protein tyrosine phosphatase 1B (PTP-1B) with an IC_50_ value of 0.06 ± 0.01 *μ*g/mL [37].

## 6. Coumarins in Clinical Trials

### 6.1. Role of Coumarin in Cardiovascular Diseases

On reviewing the ClinicalTrials.gov database, we found only 7 clinical studies registered using the keywords “coumarin” or “coumarins” (Table 2). However, on adding the term “antivitamin K,” we identified 10 publications, and the addition of “warfarin” yielded 375 registered studies.

In the last few years, modern direct oral anticoagulants (DOACs) have been generated. Instead of acting upon some factors within the coagulation cascade (like the VKAs), these drugs are targeted to a specific component of the cascade. Furthermore, DOACs show scant interaction with other medications or foods and can be administered at fixed doses without having to monitor the patient coagulation status. This allows simplification of long-term anticoagulation therapy [111].

Randomized studies have evaluated the role of oral anticoagulation with VKAs in the primary prevention of thromboembolic events in patients with nonvalvular atrial fibrillation [112–118]. One of the first of these studies was the Copenhagen AFASAK trial [113], which showed the superiority of anticoagulation with VKAs as thromboembolic prophylaxis among patients with atrial fibrillation. The research found warfarin to be more effective than aspirin or placebo in this context, with an annual stroke incidence of 2.0% (95% confidence interval [95% CI] 0.6-4.8) in the warfarin group versus 5.5% (95% CI 2.9-9.4) in the aspirin and placebo groups. The drug also reduced overall vascular mortality.

The results of the SPAF trial became known one year later [112]. This was a work involving a larger sample size and a follow-up period of 16 months. It recorded thromboembolic episodes, bleeding, and mortality and reported an annual incidence of thromboembolic events of 2.3% in the warfarin group versus 7.4% in the placebo group (relative risk [RR] = 0.67 in favour of warfarin [95% CI 0.27-0.85]; *p* = 0.01). In turn, the bleeding risk was 1.2% annually in the warfarin group and proved much lower than in the Copenhagen AFASAK trial [113], probably because of the use of less intense anticoagulation (INR 2.0-4.5). Likewise, in 1991, the CAFA (Canadian Atrial Fibrillation Anticoagulation) trial [115] compared warfarin (INR 2-3) in 187 patients versus placebo in 191 patients, with a follow-up of 16 months. The study endpoints were thromboembolic events and bleeding, and here again, the findings favoured warfarin both in terms of the number of such events and the incidence of bleeding.

Lastly, in 1992 ,the SPINAF (Stroke Prevention in Nonrheumatic Atrial Fibrillation) examination [116] contrasted low-dose warfarin (INR 1.4-2.8) versus placebo. A total of 571 males (525 in the context of primary prevention and 46 with previous CVA) were distributed to either warfarin (*n* = 281) or placebo (*n* = 290), with a follow-up period of 22-month years. The study endpoints were thromboembolic events, bleeding, and mortality. Besides the patients with no history of CVA, 19 of those administered placebos suffered thromboembolic events (annual rate 4.3%) versus only four of those administered warfarin (annual rate 0.9%) (RR = 0.79 in favour of warfarin [95% CI 0.52-0.90]; *p* = 0.001)—this observation causing early termination of the trial.

In 2016, Cannon et al. (ClinicalTrials.gov NCT 02164864) [111] studied a series of patients with atrial fibrillation subjected to coronary stent placement and anticoagulation for the prevention of CVA and stent thrombosis. The patients with atrial fibrillation subjected to stent placement were seen to have a lesser risk of bleeding when dual therapy was provided in the form of dabigatran and a P2Y12 inhibitor than when triple therapy was administered with warfarin, a P2Y12 inhibitor, and aspirin. Dual healing was not inferior to the triple-drug strategy in terms of thromboembolic risk.

About deep venous thrombosis, the EINSTEIN-DVT trial (ClinicalTrials.gov NCT00440193) [119] was an open-label study in which patients were randomized to rivaroxaban or enoxaparin, followed by a VKA agent that could be warfarin or acenocoumarol, during 3, 6, or 12 months. The sufficiency results were found to be independent of the type of VKA administered, thus suggesting that the efficacy of the two VKAs may be similar.

The coagulation system plays a crucial role in tumor development and metastasis. This has led to increasing interest in the potential benefits of anticoagulation in this field. Concerning cancer patients, several researchers [120–122] have reported a substantial risk of recurrent thrombosis in such individuals despite oral anticoagulation therapy. On comparing low molecular weight heparin (LMWH) versus coumarin, dalteparin was seen to be more active than an oral anticoagulant in risk reduction of recurrent thromboembolism, apart from incrementing bleeding risk.

Ntaios et al. [123] conducted a meta-analysis of 28 observational surveys matching the application of DOACs versus VKAs in patients with atrial fibrillation. All of the evaluated DOACs were supported to a clear and marked decrease in intracranial haemorrhage. In the latest research published by Esteve-Pastor et al. [124], non-VKA anticoagulants have been proposed as an alternative for patients with atrial fibrillation. Direct oral anticoagulants are an interesting therapeutic option, but further investigations are required before their widespread use can be recommended.

### 6.2. Studies on the Efficacy of Coumarins in Cancer Patients

Grotz et al. [125] studied the validity of combination coumarin/troxerutin therapy in protecting the salivary glands and mucous membranes of patients subjected to head and neck radiotherapy. In turn, Thornes et al. reported the efficacy of coumarin (warfarin) as adjuvant therapy in melanoma [126, 127] (Table 3).

The antitumor effect of coumarin in patients with renal cell carcinoma has been studied by administering the drug at an oral dose of 100 mg/day with the addition of cimetidine (four 300 mg doses a day from day 15 of treatment), with interesting results and practically no side effects [128, 129].

Concerning prostate cancer, patients have been treated with coumarin (3 g/day), and although the results indicated partial tumor response, the tumor burden was seen to decrease in all cases [130].

Warfarin has proved notably positive achievements in the handling of small-cell lung carcinoma. Mousa [131] found that anticoagulation with typically used drugs like nonfractionated heparin and warfarin (Coumadin®) prevents cancer development by limiting tumor cell capacity. The recent finding indicates that anticoagulant drugs and treatment with cimetidine in malignant disease can enhance patient survival and inhibit metastatic action [131].

Zacharski et al., in a phase III trial (study 75), evaluated patients with small cell and non-small-cell lung carcinoma, colon cancer, head and neck malignancies, and pancreatic cancer. The patients received chemotherapy together with warfarin or chemotherapy alone. The authors found warfarin to be associated with improved survival among the patients with small-cell lung carcinoma subjected to chemotherapy versus the patients with non-small-cell lung carcinoma (*p* = 0.018). No considerable variation was noted in the rest of the malignancies [132–134]. A randomized, prospective study of advanced small-cell lung carcinoma reported benefits in terms of tumor response and survival, though at the cost of more frequent bleeding [135]. A Norwegian population study published in JAMA Internal Medicine [136] provided data on the influence of warfarin on cancer risk. Among the subjects administered warfarin, 9.4% were diagnosed with cancer, versus 10.6% of the population not treated with warfarin. This lesser presence of malignancy in the warfarin group was particularly manifested in patients with lung cancer and atrial fibrillation, where the incidence was lower than among the rest (incidence rate ratio [IRR]: 0.39). However, in the case of colon cancer, warfarin showed no effects in terms of incidence in this population.

Lastly, a recent Cochrane review (2017) on the effects of oral VKAs in cancer patients [137] has concluded that these drugs offer no benefits in terms of survival in patients not diagnosed with thrombosis (i.e., without anticoagulation indications). Concerning the antitumor potential of VKAs (warfarin), the high associated bleeding rates have limited their use in this field. Furthermore, the negative data of the Cochrane review and the great variability of the blood levels of these drugs when administered with cytostatic agents indicate that they afford suboptimal therapeutic outcomes.

Further clinical trials are needed, with patient groups that are homogeneous in terms of the variety of cancer involved, the phase of the disease, and lifetime.

## 7. Discussion

The pharmacological activities and therapeutic applications of the simple coumarins are dependent upon their chemical substitution profile [23]. Anticancer drugs are designed to defect the abnormally dividing cell by disturbing the cell division action [142, 143]. Chemicals like DNA intercalating and cross-linking agents, topoisomerase inhibitors, cytoskeleton disrupting agents, and antimetabolites are in practice as anticancer drugs [144]. However, apart from the effectiveness of these drugs, multiple side effects have been seen (e.g., haematopoietic system). Occasionally, collective therapies are more preferably used to treat the disease which allows the least side effects during treatment. At present, surgery, chemotherapy, and radiotherapy in combination offer the best treatment for patients. Coumarin is not only effective in cancer treatment but also helps to reduce undesirable outcomes resulted in radiotherapy [65, 145].

High doses of bergapten, a coumarin found in bergamot and citrus essential oil in general, are mutagenic and carcinogenic [146]; in addition, they appear to be responsible for the inhibitory activity of the CYP3A4 isoform of cytochrome P450, characteristic of grapefruit juice (which therefore reduces the metabolism of many drugs, increasing therapeutic activity with the risk of adverse reactions from overdose) [146].

Other studies have shown that coumarin lowers glutamate levels in the hippocampus and stimulates the release of endogenous amino acids such as glutamate, taurine, and glycine in the prefrontal cortex, thus justifying the efficacy of coumarin in the treatment of neurodegenerative diseases, which are directly correlated with central nervous system imbalance. Due to its inhibitory effect on MAO, coumarins may have antidepressant properties.

Although in this updated review, some potential therapeutic effects beneficial to human health have been highlighted; there are still some limitations and clinical pitfalls that must be considered before recommending them as complementary/adjuvant treatments.

Coumarin chromen-2-one is a poisonous chemical compound found in many plants such as cinnamon, mint, green tea, and lavender [147]. In addition, this compound is the precursor of several anticoagulant drugs such as warfarin.

Coumarin derivatives such as warfarin and acenocoumarol, both of which are vitamin K antagonists (VKAs)—are widely prescribed throughout the world as oral anticoagulants to prevent and treat thromboembolic disorders. The efficacy and safety of acenocoumarol have been evaluated in atrial fibrillation, deep venous thrombosis (DVT), and heart valve replacement surgery, following acute myocardial infarction, in the postoperative period of major surgery and also in cases of prolonged hospital stay. Acenocoumarol is effective and safe in all age groups. In comparison with warfarin, it affords greater stability of the anticoagulant effect. However, treatment with coumarin derivatives remains a challenge, since the response to a given dose is characterized by great inter- and intraindividual variability. This is because certain CYP2C9 and VKORC1 polymorphisms are related to lesser dose prescription and greater bleeding risk. Acenocoumarol is inexpensive and may be an adequate oral coagulant for prolonged use. The VKAs are well tolerated and have an excellent cost-efficacy ratio. Moreover, the experience gained with the use of these drugs is very extensive, and the anticoagulation control afforded in the real-life setting is reasonable (with excellent control in the presence of patient self-control). On the other hand, the VKAs have a universally available antidote (vitamin K) that is very easy to use and inexpensive. In general, VKAs pose a slightly increased risk of intracranial haemorrhage, though a probably lesser risk of gastrointestinal bleeding compared with other drugs [148–151]. Optimum anticoagulation should seek a proportion among the prohibition of cerebrovascular accidents (CVAs) and the appearance of bleeding complications. In this respect, standardized VKA therapy based on an international normalized ratio (INR) of 2-3 is effective.

On the other hand, VKAs have well-known limitations, including a narrow therapeutic window, easy access and disappearance of effect, numerous pharmacological and food interactions, unpredictable pharmacokinetics (great inter- and intraindividual variability in response to a given drug dose, as commented above), and the need for periodic laboratory test monitoring with frequent dose adjustments that complicate treatment and interfere with patient quality of life [23, 148–151].

Natural coumarins have also a casein kinase-2 (CK2) inhibitory activity. CK2 is perhaps the best-known pleiotropic protein kinases which have greater than the three hundred recognizable protein substrate, and this could be the probable reason for its fermentative action. The two enzymatic subunits are effective in either presence or absence of regulatory subunits. However, the active CK2 is universal, crucial, and associated in a diverse range of imperative cell functions, but evidence suggested that its enzymatic subunits can also act as oncogenes. It is observed that they show an antiapoptotic influence in prostatic carcinoma cell lines. Coumarin-linked piperazine is considered the most promising inhibitor of CK2 [152]. The experiment showed that this inhibitor crystalized in complex with CK2, and the trial binding mode has been used to derive the linear interaction energy (LIE) model. In the past several years, multiple testing programs have been done by employing both *in silico* and conventional methods to discover novel powerful and electively CK2 blocking agents.

Particular attention should be paid to the use of dried plants containing coumarin, due to the already mentioned capacity to produce dicumarol in certain situations. These natural compounds are contraindicated in patients undergoing treatment with coumarin anticoagulants such as sintrom or antiplatelet agents (aspirin and clopidogrel).

Although the existing clinical trials are limited, their findings suggest a positive impact of anticoagulation in cancer patients. Nevertheless, this advantage is not the same in all cases, since some histological types of tumor appear to be extra responsive to the action of anticoagulation therapy than others in the early stages of neoplastic development [120–122].

Due to their photosensitizing properties, 6, 7-furano-coumarins are contraindicated in case of prolonged exposure to sunlight, due to the risk of photodermatitis, burns, and melanomas [153]. Doses of coumarin in a human's daily diet were calculated at 0.02 mg/bw, and the concentration of coumarin varies from 1 m/bw in celery to 7000 mg/bw in Ceylon cinnamon and over 87000 mg/bw in Cassia cinnamon. Therefore, the association of nutritional supplements containing coumarins with these plants should be avoided [14]. Due to the proven hepatotoxicity in experimental studies in rats, FDA has banned the use of coumarin as a food additive [154]. However, adverse events in humans after natural coumarin administration were rare.

## 8. Concluding Remarks and Perspectives

Coumarins belong to the benzopyrone family widespread in nature. Natural coumarins demonstrated a wide spectrum of pharmacological activities, including anti-inflammatory, anticoagulant, anticancer, antibacterial, antimalarial, casein kinase-2 (CK2) inhibitory, antifungal, antiviral, Alzheimer's disease inhibition, neuroprotective, anticonvulsant, phytoalexins, ulcerogenic, and antihypertensive. There are very few studies on the bioavailability of coumarins; therefore, extra investigations are required to study the bioavailability of different coumarins which already showed good biological activities in previous studies.

Based on the wide range of pharmacological effects, this review presents a common analysis of the origin, availability, and biological capacities of coumarins with a further discussion on the most crucial mode of their therapeutic actions. The coumarins play an important task in different pharmacological pathways and make a significant contribution to the development of new therapeutic targets. In conclusion, a broad variety of coumarins are available, and their pharmacological activities are of current interest thanks to their synthetic accessibility and richness in plants and other natural objects. Coumarins play an essential role as therapeutic agents in a range of medical fields.

## Figures and Tables

**Table 1:** Summarized biological activities of different type of coumarins.

Coumarin type	Example	Pharmacological activities
Simple coumarins	Coumarin [33] 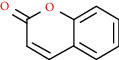	Anti-inflammatory
	Esculetin [34] 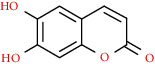	Antioxidant Anti-inflammatory Anticancer Neuroprotective
	Ammoresinol [35] 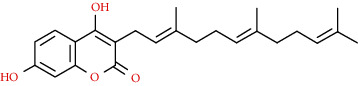	Antibacterial
	Ostruthin [35] 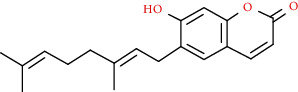	Antimicrobial
	Osthole [36, 37] 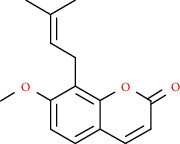	Antioxidant Antimicrobial Antitumor Anticonvulsant Antidiabetic
	Novobiocin [38] 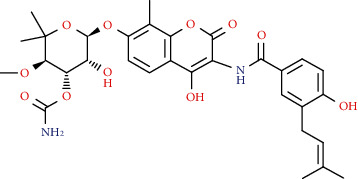	Antibacterial
	Coumermycin [39] 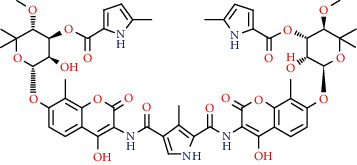	Antibacterial
	Chartreusin [40] 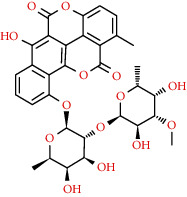	Antibacterial Anticancer
	Fraxin [41] 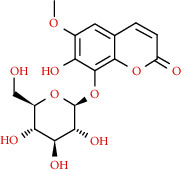	Antioxidant Anticancer Antiadipogenic
	Umbelliferone [42] 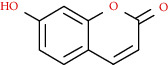	Antitubercular
	Fraxidin [43] 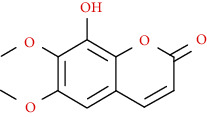	Antiadipogenic Antihyperglycemic
	Phellodenol A [44] 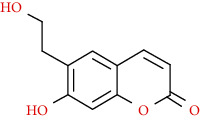	Antitubercular
	Esculin [45] 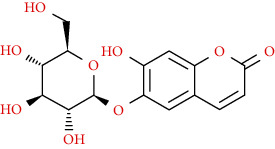	Antiadipogenic

Furano coumarins	Imperatorin [46] 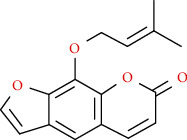	Anti-inflammatory Antimicrobial Anticancer Anticonvulsant
	Psoralen [17] 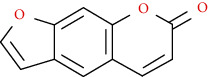	Antifungal Antituberculosis
	Bergapten [45] 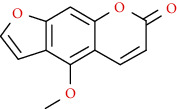	Antituberculosis
	Methoxsalen [47] 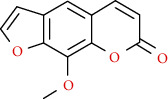	Cytochrome P450 inhibitor

Dihydrofurano coumarins	Anthogenol [48] 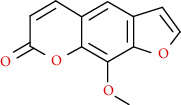 Felamidin [49] 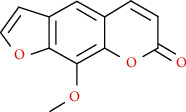 Marmesin [42] 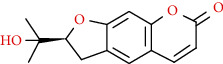	Antibacterial Antibacterial Antituberculosis

Linear type	Agasyllin [50] Aegelinol benzoate [50] Xanthyletin [42] 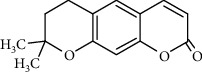	Antibacterial Antibacterial Antituberculosis

Angular type	Inophyllum A, B, C, E, P, G1, and G2 [51] 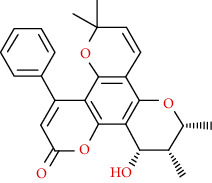 Calanolide A, B, and F [52] 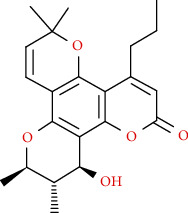 (+)-Dihydrocalanolide A and B [53] 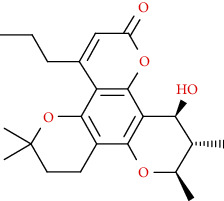 Pseudocordatolide C [54] 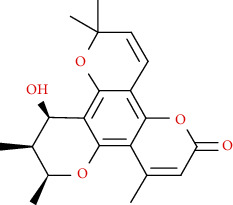	Antiviral Antiviral Antiviral Antiviral

Bicoumarins	Dicumarol [55] 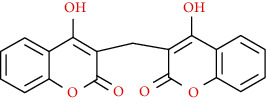	Anticoagulant

**Table 2:** Registered coumarin clinical trials on the Clinicaltrials.gov database.

Title	Participants	Type of trial	Period	Outcomes
Effectiveness of a Multidisciplinary Medication Review with Follow-up for Patients Treated with Coumarin Anticoagulants in Primary Care *NCT03154489*	204	Randomized	May 2017-May 2018	Effectiveness of medication review with follow-up. Principally studying INR control of these patients during 6 months
Efficacy and Safety of Coumarin and Troxerutin in the symptomatic Treatment of Chronic Venous Insufficiency *NCT01848210*	829	Randomized	May 2013-September 2015	Mean Change (Reduction) from Baseline in Volume of Reference Le g at Week 16 Change in the partial volume of legs will be measured using a water plethysmometer.
Efficacy and Safety Study of BERIPLEX® P/N (Kcentra) Compared with Plasma in Patients with Acute Major Bleeding Caused by Anticoagulant Therapy *NCT00708435*	216	Randomized	-	Percentage of participants achieving hemostatic efficacy of stopping an ongoing major bleed: at 1 and 4 hours after the end of infusion Percentage of participants who had a rapid decrease of the international normalized ratio (INR) time frame: 30 minutes after the end of infusion
Anticoagulation in Liver Fibrosis *NCT00180674*	12	Nonrandomized	August 2005-October 2006	-
An Open-label, Randomized, Multicenter Phase IIIb Study to Assess the Efficacy, Safety and Tolerance of BERIPLEX® P/N (Kcentra) Compared with Plasma for Rapid Reversal of Coagulopathy Induced by Vitamin K Antagonists in Subjects Requiring an Urgent Surgical Procedure (BE1116_3003) *NCT00803101*	176	Randomized	February 2009-February 2013	Percentage of participants achieving hemostatic efficacy during surgery the start of infusion until the end of surgery Percentage of participants who had a rapid decrease of the INR: 30 minutes after the end of infusion
Therapeutic Equivalence between Branded and Generic WARFArin Tablets in Brazil (WARFA) *NCT02017197*	100	Randomized	August 2014-August 2016	Difference between Delta INR at the fourth week of each period
Rivaroxaban Compared to Vitamin K Antagonist upon Development of Cardiovascular Calcification *NCT02066662*	192	Randomized	July 2013-active	Progression of coronary and aortic valve calcification (Agatston, volume & mass score as assessed by cardiac CT)

**Table 3:** Clinical studies with warfarin in treating cancers.

Cancer [Ref.]	Intervention	Study design	Observation
Colorectal cancer [138]	Warfarin for 24 months vs. control	RCTa, unblinded	No significant difference (72.2% in the warfarin group vs. 69.5% in the control group)
Breast cancer [139]	Warfarin during chemotherapy vs. placebo	RCTa, double-blind	Insignificant variation for 225-day survival (57% in warfarin group vs. 63% in the placebo group)
Lung cancer [140] [132] [141]	Warfarin life-long vs. control Warfarin life-long vs. control Warfarin life-long vs. control	RCTa, unblinded RCTa, unblinded RCTa, unblinded	Insignificant variation for median survival (9.7 months in the warfarin group vs. 8.9 months in the control) Significant increase in median survival (49.5 weeks in warfarin group vs. 23 weeks in control) No significant difference in median survival
Melanoma stage Ib and II melanoma [126]	Warfarin for 24 months after surgery vs. placebo	RCTa, double-blind	Significant decrease in recurrence (4/13 in warfarin group vs. 10/14 in the placebo group)
Advanced head and neck cancer [133]	Warfarin life-long vs. control	RCTa, unblinded	No significant difference in median survival (18.0 weeks in the warfarin group vs. 20.7 weeks in the control group)

## Data Availability

The data used to support the findings of this study are available from the corresponding author upon request.
